# 气相色谱-电子捕获负化学源-低分辨质谱法测定人体血液不同组分中短链及中链氯化石蜡

**DOI:** 10.3724/SP.J.1123.2022.11012

**Published:** 2023-08-08

**Authors:** Shuang YU, Yuan GAO, Xiuhua ZHU, Ningbo GENG, Yubing DAI, Jianyao HONG, Jiping CHEN

**Affiliations:** 1.中国科学院分离分析化学重点实验室,中国科学院大连化学物理研究所,辽宁 大连 116023; 1. CAS Key Laboratory of Separation Science for Analytical Chemistry, Dalian Institute of Chemical Physics, Dalian 116023, China; 2.大连交通大学环境与化学工程学院,辽宁 大连 116028; 2. School of Environmental and Chemical Engineering, Dalian Jiaotong University, Dalian 116028, China

**Keywords:** 气相色谱-电子捕获负化学源-低分辨质谱法, 短链氯化石蜡, 中链氯化石蜡, 样品提取与净化, gas chromatography-electron capture negative ion-low resolution mass spectrometry (GC-ECNI-LRMS), short-chain chlorinated paraffins (SCCPs), medium-chain chlorinated paraffins (MCCPs), sample extraction and purification

## Abstract

短链及中链氯化石蜡(SCCPs和MCCPs)因其持久性、生物毒性、富集性及长距离迁移等特点受到广泛关注。随着SCCPs和MCCPs在多种环境介质中的广泛检出,人类面临的暴露风险逐渐升高,因此开展人体内SCCPs和MCCPs的暴露评估具有重要意义。了解氯化石蜡(CPs)及其各同族体在血浆和血细胞中的分配行为及分布特征能够更好地掌握其在人体内的积累和循环情况,有助于准确、有效地评估CPs在人体内的暴露水平。本研究建立了Percoll不连续密度梯度离心方法,将人体血液分离为血浆、红细胞、白细胞和血小板4种组分,利用超声进行细胞破碎和提取,并采用多层硅胶柱去除脂质干扰。结果表明,使用80 mL正己烷-二氯甲烷(1∶1, v/v)和50 mL二氯甲烷作为样品净化的连续洗脱溶剂(合并收集)可实现血液样品中CPs与脂类大分子的选择性分离。采用气相色谱-电子捕获负化学源-低分辨质谱法(GC-ECNI-LRMS)测定人体血液不同组分中的SCCPs和MCCPs, SCCPs和MCCPs的方法检出限分别为1.57 ng/g湿重(*n*=7)和8.29 ng/g湿重(*n*=7),提取内标的回收率分别为67.0%~126.6%和69.5%~120.5%。采用该方法对采集的人体血液样品进行分析,所有样品中均有SCCPs和MCCPs检出,检出含量分别为10.81~65.23 ng/g湿重和31.82~105.65 ng/g湿重,其中红细胞中的SCCPs和MCCPs含量最高,其次是血浆,白细胞和血小板中的CPs含量相对较低。SCCPs和MCCPs在人体血液不同组分中的分布模式相似,SCCPs以C_10_-CPs为主,MCCPs以C_14_-CPs为主。该方法灵敏度高,操作便捷,能够满足人体血液样品的组分分离及各组分中SCCPs和MCCPs的定量分析需求。

## Abstract (en)

Short- and medium-chain chlorinated paraffins (SCCPs and MCCPs) have attracted significant attention because of their persistence, biotoxicity, bioaccumulation, and long-range migration. Given their worldwide detection in a variety of environmental matrices, concerns related to the high exposure risks of SCCPs and MCCPs to humans have grown. Thus, knowledge of the contamination patterns of SCCPs and MCCPs and their distribution characteristics in the vivo exposure of humans is of great importance. However, little information is available on the contamination of SCCPs and MCCPs in human blood/plasma/serum, mainly because of the difficulty of sample preparation and quantitative analysis. In this study, a new blood sample pretreatment method based on Percoll discontinuous density gradient centrifugation was developed to separate plasma, red blood cells, white blood cells, and platelets from human whole blood. A series of Percoll sodium chloride buffer solutions with mass concentrations of 1.095, 1.077, and 1.060 g/mL were placed in a centrifuge tube from top to bottom to establish discontinuous density gradients. The dosage for each density gradient was 1.5 mL. Human whole blood samples mixed with 0.85% sodium chloride aqueous solution were then added to the top layer of the Percoll sodium chloride solution. After centrifugation, the whole blood was separated into four components. The plasma was located at the top layer of the centrifuge tube, whereas the platelets, white blood cells, and red blood cells were retained at the junction of the various Percoll sodium chloride solutions. The sampling volume of human whole blood and incubation time were optimized, and results indicated that an excessively long incubation time could lead to hemolysis, resulting in a decrease in the recoveries of SCCPs and MCCPs. Therefore, a sampling volume of 1.5 mL and incubation time of 10 min at 4 ℃ were adopted. The cells of the blood components were further broken and extracted by ultrasonic pretreatment, followed by multilayer silica gel column chromatography for lipid removal. The use of 80 mL of *n*-hexane-dichloromethane (1∶1, v/v) and 50 mL of dichloromethane as the elution solvents (collected together) for the gel column separated the SCCPs and MCCPs from the lipid molecules in the blood samples. Gas chromatography-electron capture negative ion-low resolution mass spectrometry (GC-ECNI-LRMS) was used to determine the SCCPs and MCCPs. Quantification using the corrected total response factor with degrees of chlorination was achieved with linear corrections (*R*^2^=0.912 and 0.929 for the SCCPs and MCCPs, respectively). The method detection limits (MDLs) for the SCCPs and MCCPs were 1.57 and 8.29 ng/g wet weight (ww, *n*=7), respectively. The extraction internal standard recoveries were 67.0%-126.6% for the SCCPs and 69.5%-120.5% for the MCCPs. The developed method was applied to determine SCCPs and MCCPs in actual human whole blood samples. The contents of SCCPs and MCCPs were 10.81-65.23 and 31.82-105.65 ng/g (ww), respectively. Red blood cells exhibited the highest contents of CPs, followed by plasma, white blood cells, and platelets. The proportions of SCCPs and MCCPs in red blood cells and plasma were 70% and 66%, respectively. In all four components, the MCCP contents were higher than the SCCP contents, and the ratios of MCCPs to SCCPs ranged from 1.04 to 3.78. Similar congener patterns of SCCPs and MCCPs were found in the four components of human whole blood. C_10_-CPs and C_14_-CPs were predominantly observed in the SCCPs and MCCPs, respectively. In summary, a simple and efficient method was proposed to determine low concentrations of SCCPs and MCCPs in human blood with high sensitivity and selectivity. This method can meet requirements for the quantitative analysis of SCCPs and MCCPs in human blood components, thereby providing technical support for human health risk assessment.

氯化石蜡(chlorinated paraffins, CPs)是一种正构烷烃的氯代衍生物,其碳链长度为10~30个碳原子,氯化程度为30%~72%,它们是由不同碳链长度的正构烷烃或其混合物在紫外光或高温下的非选择性氯化反应产生的^[1]^。CPs根据碳链长度可分为短链氯化石蜡(SCCPs,C_10_~C_13_)、中链氯化石蜡(MCCPs,C_14_~C_17_)和长链氯化石蜡(LCCPs,C_18_~C_30_)^[2]^。在20世纪30年代初,CPs由于其具有极耐压性能被用于金属加工液中,目前它们被广泛用作阻燃剂和聚氯乙烯(PVC)增塑剂,用于生产电缆、地板、软管、人造皮革和橡胶产品,以及作为油漆、润滑剂和其他工业产品的添加剂^[3]^。我国从20世纪50年代末开始生产CPs,自2000年起,我国成为全球CPs的主要生产国与消费国;2010年,欧美国家停止生产SCCPs,转而使用MCCPs和LCCPs,但全球总CPs产量仍保持上升趋势^[4,5]^。CPs不仅产量高、环境释放量大,而且其可以通过不同暴露途径对环境和人体健康造成潜在威胁,因而受到了国际社会的高度关注。已有研究表明,SCCPs具有持久性、生物毒性、生物富集性和长距离迁移等特点,对环境和人类健康会产生危害,并于2017年5月被列入《关于持久性有机污染物的斯德哥尔摩公约》附件A^[6,7]^。MCCPs作为SCCPs的替代品,其在世界各地的产量均有所增长,然而由于MCCPs具有与SCCPs相似的结构和物理化学特性,2022年1月英国提议将氯化程度≥45%的MCCPs列入《关于持久性有机污染物的斯德哥尔摩公约》候选物质清单^[8]^。

SCCPs对水生生物具有较高毒性,对斑马鱼胚胎中的甘油磷脂代谢、脂肪酸代谢、嘌呤代谢和氨基酸代谢等具有显著的影响^[9,10]^; SCCPs对虹鳟鱼的慢性毒性主要表现为肝组织损伤^[11]^;在啮齿动物的毒性研究中,SCCPs对大鼠及小鼠的甲状腺、肾脏和淋巴瘤细胞具有致癌性和致突变性能^[12⇓-14]^。目前尚无关于CPs对人体直接产生毒性的报道,然而研究发现SCCPs对人体肝癌细胞(HepG2)代谢具有显著影响。MCCPs的毒性效应研究较少,尽管目前没有数据显示MCCPs具有致癌性,但是其健康风险和毒性效应也不容忽视。

随着SCCPs和MCCPs在多种环境介质中的广泛检出,人类面临的SCCPs和MCCPs暴露风险升高,评估其在人体内的暴露水平十分必要。Zhou等^[15]^使用高效液相色谱-电喷雾电离-四极杆飞行时间质谱法同时测定了人体血清中SCCPs和MCCPs的水平。Ding等^[16]^使用氯增强大气压电离源-四极杆飞行时间质谱法测定我国山东省济南市50~84岁居民血清中SCCPs和MCCPs的水平和同系物分布。Xu等^[17]^分析了大连地区人群血浆中的SCCPs含量,为12.6~203 ng/g。目前,人体样品的生物监测研究较少,且主要集中在血清或血浆基质中,然而CPs在血液不同组分间的分配还未见报道,SCCPs和MCCPs各同族体在血液间的分配行为也不清楚。了解CPs及其各同族体在血浆和血细胞间的分配行为和分布特征能更好地掌握CPs在人体内的积累和循环情况,有助于准确、有效地评估人体内暴露水平。

本研究采用气相色谱-电子捕获负化学源-低分辨质谱法(GC-ECNI-LRMS)检测人体血液不同组分(包括红细胞(RBC)、血浆(plasma)、白细胞(WBC)、血小板(platelet))中SCCPs和MCCPs的水平及同族体分布。采用不同浓度梯度的Percoll细胞分离液将人体血液分离为血浆、血小板、白细胞、红细胞,利用超声进行细胞破碎和提取,并用多层硅胶柱去除脂质干扰。基于GC-ECNI-LRMS分析方法,通过优化样品制备过程、实施质量保证和质量控制,建立了一种可靠的人体血液不同组分中SCCPs和MCCPs的检测方法,并对该方法进行评价。

## 1 实验部分

### 1.1 仪器、试剂与材料

气相色谱-电子捕获负化学源-低分辨质谱(GCMS-QP2010,日本Shimadzu公司);超声细胞破碎仪(JY96-IIN,宁波新芝生物科技股份有限公司);数控超声清洗器(KQ-250DE型,昆山市超声仪器有限公司);涡旋混合器(XW-80A,上海精科实业有限公司);旋转蒸发仪(R-205,瑞士BUCHI公司);台式离心机(D-37520 Osterode,德国Kendro公司);氮吹浓缩仪(DC-12,上海安普公司); Milli-Q超纯水仪(德国Merck Millipore公司)。

100 ng/μL SCCPs标准溶液(氯含量分别为51.5%、55.5%、63%,溶于环己烷中)和100 ng/μL MCCPs标准溶液(氯含量分别为42%、52%、57%,溶于环己烷中)均购自德国Dr. Ehrenstorfer公司,^13^C标记的反式氯丹(^13^C_6_-*trans*-CD)和六氯苯(^13^C_6_-HCB)均购自美国Cambridge Isotope Laboratories公司。

正己烷、二氯甲烷(农残级,美国J. T. Baker公司);壬烷(色谱纯,德国百灵威公司);无水硫酸钠、氢氧化钾(分析纯,天津市大茂化学试剂厂);浓硫酸(优级纯,天津市科密欧化学试剂有限公司);硅胶(100~200目,青岛恒泽硅胶制药有限公司);胎牛血清(fetal bovine serum, FBS,浙江天杭生物科技有限公司); Percoll细胞分离液(1.131 g/mL,北京索莱宝科技有限公司)。

无水硫酸钠和活性硅胶:在使用前,将无水硫酸钠和活性硅胶于650 ℃在马弗炉中活化4 h,冷却后密封保存于干燥器中;44%(质量分数)酸化硅胶:将200 g活性硅胶和157 g浓硫酸放于棕色瓶内,充分振荡2~3 h后,置于干燥器中保存;2%(质量分数)碱性硅胶:向100 g硅胶中加入40 mL氢氧化钾溶液(50 g/L),充分搅拌,在50~80 ℃下减压脱水至粉末状,置于干燥器中保存。

### 1.2 标准溶液的配制

将氯含量为51.5%和55.5%的SCCPs标准溶液等体积混合,得到氯含量为53.5%的SCCPs混合标准溶液;将氯含量为55.5%和63%的SCCPs标准溶液等体积混合,得到氯含量为59%的SCCPs混合标准溶液;将氯含量为42%和52%的MCCPs标准溶液等体积混合,得到氯含量为47%的MCCPs混合标准溶液;将氯含量为52%和57%的MCCPs标准溶液等体积混合,得到氯含量为54.5%的MCCPs混合标准溶液。所有混合标准溶液置于4 ℃冰箱中冷藏保存。

将^13^C_6_-*trans*-CD和^13^C_6_-HCB分别作为提取内标和进样内标,用壬烷配制成质量浓度分别为10 ng/μL和1 ng/mL的标准溶液,置于4 ℃冰箱中冷藏保存。

### 1.3 血液样品采集

采集本地孕妇血液样品(由大连医科大学附属第二医院提供),将样品保存于真空采血管(含抗凝剂)中,采集完毕后尽快送至实验室,运输全程低温保存,采样时间为2021年11月。

### 1.4 样品前处理

将血液样品通过不同浓度梯度的Percoll氯化钠缓冲液分离为血浆、血小板、白细胞、红细胞4种血液基质,分离后的样品在-80 ℃冰箱中冷冻保存,待分析。将冷冻后的血液样品转移至4 ℃冰箱中进行解冻;将血浆、血小板、白细胞、红细胞分别转移至用正己烷多次润洗的玻璃离心管中,加入超纯水定容至一定体积后,在冰水浴中进行超声细胞破碎,超声破碎条件为每隔10 s超声10 s,时长共1 min;随后添加提取内标,以10 mL正己烷-二氯甲烷(1∶1, v/v)作为提取溶剂,超声提取3次,每次超声10 min;超声后经高速离心获得有机相上清液,合并上清液并浓缩至1~2 mL,待净化。

采用多层硅胶柱净化,自下至上依次装填5 g无水硫酸钠、2 g 2%碱性硅胶、2 g活性硅胶、5 g 44%酸化硅胶和6 g无水硫酸钠。将待净化样品完全转移至多层硅胶柱,先用50 mL正己烷淋洗以去除杂质,再用80 mL正己烷-二氯甲烷(1∶1, v/v)和50 mL二氯甲烷连续洗脱,合并收集洗脱液。将洗脱液通过旋转蒸发浓缩至1~2 mL,氮气吹扫浓缩至近干后,加入进样内标^13^C_6_-HCB,涡旋混匀后待上机分析。

### 1.5 质量控制与质量保证

所有玻璃器皿使用专用洗涤液进行清洗后,用超纯水冲洗3次,使用前再用正己烷润洗3次。空白实验:将2 mL超纯水加入到玻璃离心管中,再依据以上处理步骤进行样品前处理及仪器分析,每6个血液基质样品插入一个空白样品,以监测实验过程中可能产生的污染。实验过程中所用溶剂、填料均做空白对照实验,空白样品中SCCPs和MCCPs的浓度均低于方法检出限。

### 1.6 仪器分析

#### 1.6.1 色谱条件

毛细管气相色谱柱为DB-5M毛细管柱(15 m×0.25 mm×0.25 μm, J&W Scientific, USA);进样口温度为280 ℃,不分流进样,进样量1 μL。程序升温条件如下:初始100 ℃保持2 min;以20 ℃/min升温至160 ℃,保持2 min;再以30 ℃/min升温至310 ℃,保持12 min。

#### 1.6.2 质谱条件

质谱离子源为电子捕获负化学源,气相色谱和质谱的接口传输线温度为280 ℃,离子源温度为200 ℃,以甲烷作为反应气,流速为2 mL/min。在选择离子模式(SIM)下检测SCCPs和MCCPs相对丰度最高的[M-Cl]^-^碎片离子(表1),其中[M-Cl]^-^同位素丰度最高的碎片离子作为定量离子,相对丰度次高的碎片离子作为定性离子。以[M]^-^作为^13^C_6_-*trans*-CD和^13^C_6_-HCB的定量离子。

**表1:** SCCPs和MCCPs同族体的定量离子和定性离子

SCCPs					MCCPs				
Formula	Chlorine content/%	Quantitative ion (*m/z*)	Qualitative ion (*m/z*)		Formula		Chlorine content/%	Quantitative ion (*m/z*)	Qualitative ion (*m/z*)
C_10_H_17_Cl_5_	56.4	279	277			C_14_H_25_Cl_5_	47.80	333	335
C_10_H_16_Cl_6_	61.0	313	315			C_14_H_24_Cl_6_	52.50	369	371
C_10_H_15_Cl_7_	64.8	347	349			C_14_H_23_Cl_7_	56.50	403	405
C_10_H_14_Cl_8_	67.9	381	383			C_14_H_22_Cl_8_	59.80	437	439
C_10_H_13_Cl_9_	70.6	417	415			C_14_H_21_Cl_9_	62.80	473	471
C_10_H_12_Cl_10_	72.9	451	449			C_14_H_20_Cl_10_	65.30	505	507
C_11_H_19_Cl_5_	54.0	293	291			C_15_H_27_Cl_5_	46.10	349	347
C_11_H_18_Cl_6_	58.7	327	329			C_15_H_26_Cl_6_	50.80	383	385
C_11_H_17_Cl_7_	62.5	361	363			C_15_H_25_Cl_7_	54.70	417	419
C_11_H_16_Cl_8_	65.7	395	397			C_15_H_24_Cl_8_	58.10	451	453
C_11_H_15_Cl_9_	68.5	431	429			C_15_H_23_Cl_9_	61.10	485	487
C_11_H_14_Cl_10_	70.9	465	463			C_15_H_22_Cl_10_	63.70	521	519
C_12_H_21_Cl_5_	51.8	307	305			C_16_H_29_Cl_5_	44.50	361	363
C_12_H_20_Cl_6_	56.5	341	343			C_16_H_28_Cl_6_	49.10	397	399
C_12_H_19_Cl_7_	60.4	375	377			C_16_H_27_Cl_7_	53.10	431	433
C_12_H_18_Cl_8_	63.7	409	411			C_16_H_26_Cl_8_	56.50	465	467
C_12_H_17_Cl_9_	66.5	445	443			C_16_H_25_Cl_9_	59.50	501	499
C_12_H_16_Cl_10_	68.9	479	477			C_16_H_24_Cl_10_	62.10	535	533
C_13_H_23_Cl_5_	49.8	321	319			C_17_H_31_Cl_5_	43.00	375	377
C_13_H_22_Cl_6_	54.5	355	357			C_17_H_30_Cl_6_	47.60	411	413
C_13_H_21_Cl_7_	58.4	389	391			C_17_H_29_Cl_7_	51.50	443	445
C_13_H_20_Cl_8_	61.7	423	425			C_17_H_28_Cl_8_	55.00	477	479
C_13_H_19_Cl_9_	64.6	459	457			C_17_H_27_Cl_9_	58.00	515	513
C_13_H_18_Cl_10_	67.1	493	491			C_17_H_26_Cl_10_	60.60	549	547

## 2 结果与讨论

### 2.1 Percoll不连续密度梯度离心方法的建立

血液是由血浆和几种不同种类的血细胞组成的组织,其中血浆占55%左右,血细胞如红细胞、血小板和白细胞约占血液容量的45%。血液中不同组分的分离有助于深入了解污染物在人体血液不同组分间的分配和准确评估人体暴露水平及污染物对人体的毒性效应。目前分离血细胞所用的方法有荧光激活细胞分选、亲和柱色谱法和膜过滤等方法,但由于器械体积大、操作难度和成本高、耗时较长等缺点,这些技术的应用均受到不同程度的限制^[18]^。

Percoll是经过聚乙烯吡咯烷酮(PVP)处理的硅胶颗粒混悬液,无细胞毒性和刺激性,常应用在细胞分离中。首先,配制系列浓度梯度的Percoll氯化钠缓冲溶液,质量浓度依次为1.095、1.077、1.060 g/mL,按质量浓度从高到低依次逐层铺设在康宁离心管中以建立不连续密度梯度,每个密度梯度用量为1.5 mL,离心管在使用前用FBS进行润洗;之后用0.85%氯化钠水溶液将人体血液样品稀释混匀,加入到Percoll氯化钠缓冲溶液的最上层,待其逐层通过;之后将离心管于22 ℃、400 g下离心5 min后,再于800 g下离心15 min。离心后血浆位于离心管的最顶层,其余3种血细胞因密度差异分别停留在相邻浓度梯度的Percoll氯化钠缓冲溶液的交界处,从上至下分别为血小板层、白细胞层和红细胞层(图1),实现了血液样品不同组分间的分离。

**图1:**
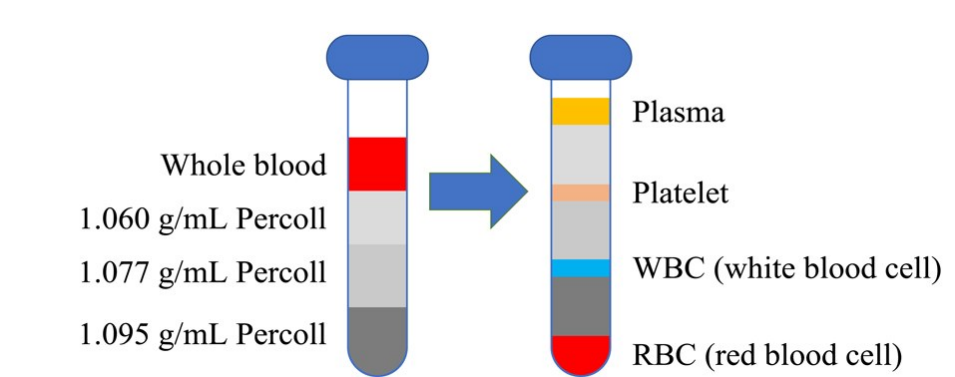
Percoll不连续密度梯度离心法分离人体全血样品的示意图

### 2.2 洗脱溶剂的选择

分离后的不同组分血液样品经超声提取后采用多层硅胶柱净化,分别考察了单独使用正己烷-二氯甲烷(1∶1, v/v)洗脱、正己烷-二氯甲烷(1∶1, v/v)和二氯甲烷连续洗脱对SCCPs和MCCPs回收率的影响。当采用80 mL正己烷-二氯甲烷(1∶1, v/v)洗脱时,血浆、血小板、白细胞、红细胞中SCCPs和MCCPs的回收率分别为60%~67%和60%~87%,如图2a所示;当采用80 mL正己烷-二氯甲烷(1∶1, v/v)和50 mL二氯甲烷溶液连续洗脱时,血浆、血小板、白细胞和红细胞中SCCPs的回收率可达到73%以上,MCCPs的回收率为88%~93%,如图2b所示。结果表明,连续洗脱可提高血液不同组分样品中SCCPs和MCCPs的回收率。为确保目标物能够洗脱完全,最终采用80 mL正己烷-二氯甲烷(1∶1, v/v)和50 mL二氯甲烷作为样品净化的洗脱溶剂。

**图2:**
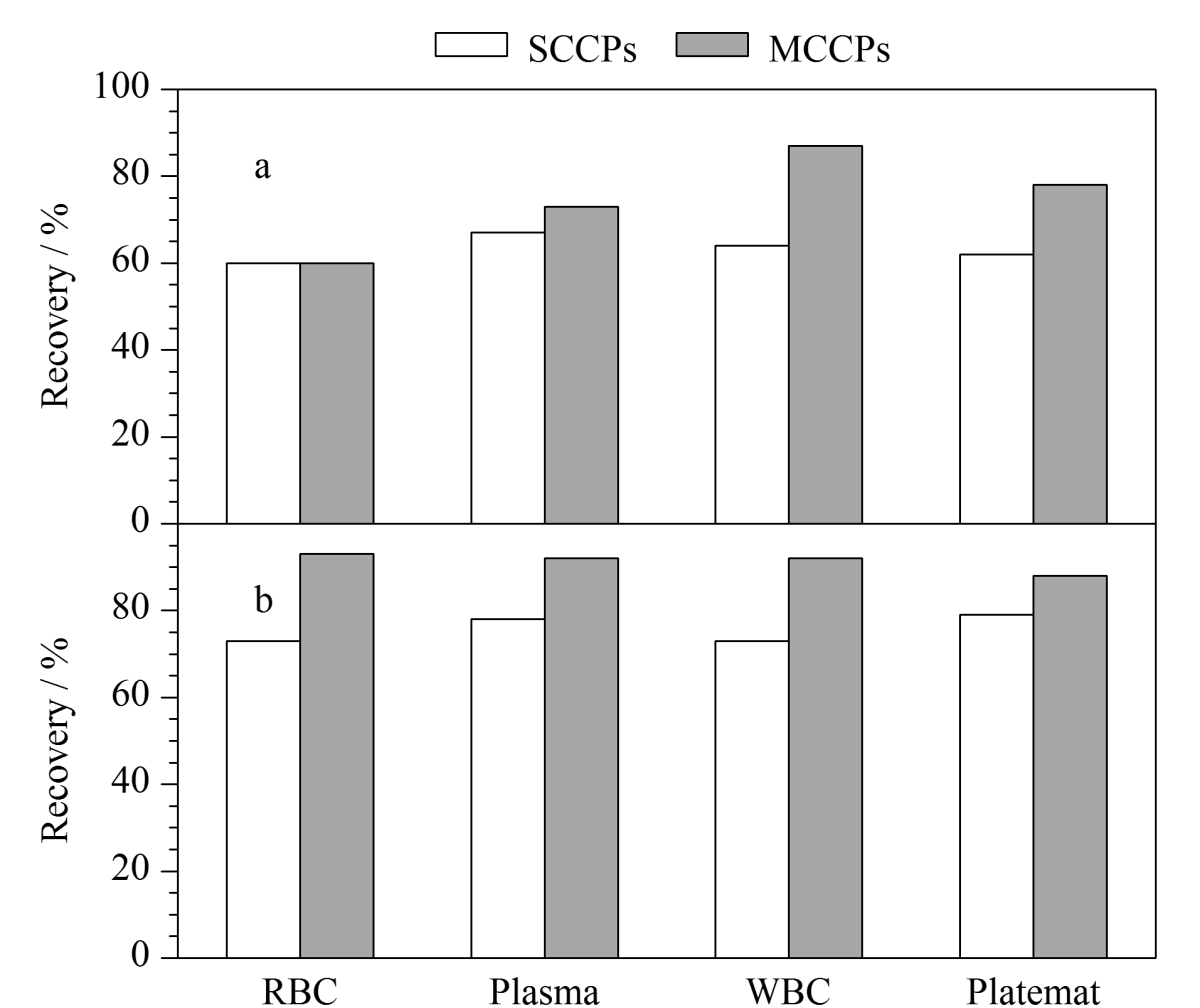
(a)采用80 mL正己烷-二氯甲烷(1∶1, v/v)洗脱与(b)采用80 mL正己烷-二氯甲烷(1∶1, v/v)和50 mL二氯甲烷连续洗脱对SCCPs和MCCPs回收率的影响

### 2.3 样品体积的优化

为了确定人体血液样品用量,将氯含量为55.5%的SCCPs标准溶液分别加入到不同体积(1.5、3.0、4.5 mL)的人体血液样品中,通过评估SCCPs的回收率来优化样品体积。取10 μL SCCPs标准溶液(100 ng/μL,环己烷),用氮吹仪快速吹干后加入一定体积的二甲亚砜(DMSO)进行定容,其中DMSO的添加体积不能超过血液样品体积的0.5%。对于不同体积(1.5、3.0、4.5 mL)的人体血液样品分别采用7、10、20 μL DMSO定容,用滴度板摇床轻微振荡进行孵育,孵育过程如表2所示。结果表明,振荡时间过长容易造成溶血现象,导致回收率下降,如4.5 mL人体血液样品在振荡4 h后出现溶血现象,血浆、血小板、白细胞、红细胞中SCCPs的回收率较低,均≤44.9%。对于3.0 mL和1.5 mL人体血液样品均轻微振荡10 min,然后在4 ℃下静置12 h。当样品体积为3.0 mL时,SCCPs在血液样品中的回收率波动较大,为49.3%~84.5%;当样品体积为1.5 mL时,SCCPs的回收率保持在72.3%~94.3%。因此后续分析均采用1.5 mL血液样品,经轻微振荡10 min后在4 ℃下静置12 h。

**表2:** 人体血液样品体积对SCCPs回收率的影响

Sample volume/mL	DMSO volume/μL	Incubation period	Recovery/%
4.5	20	slight shake for 4 h	≤44.9
3.0	10	slight shake for 10 min, keep stationary at 4 ℃ for 12 h	49.3-84.5
1.5	7	slight shake for 10 min, keep stationary at 4 ℃ for 12 h	72.3-94.3

DMSO: dimethyl sulfoxide.

### 2.4 线性范围、方法检出限和精密度

本实验以SCCPs和MCCPs标准溶液的保留时间、质荷比(*m/z*)以及色谱峰形作为参照标准,采用Reth等^[21]^提出的氯含量校正总响应因子定量方法进行分析。首先分别对5种不同氯含量的SCCPs和MCCPs标准溶液进行分析计算得到SCCPs、MCCPs的总响应因子(*y*_1_、*y*_2_)和对应的氯含量(*x*_1_、*x*_2_),对二者进行线性回归,得到回归方程*y*_1_=1.6919*x*_1_-0.9843和*y*_2_=1.9935*x*_2_-1.0285。SCCPs和MCCPs的总响应因子与氯含量呈线性相关,相关系数(*R*^2^)分别为0.912和0.929。

通过计算人体血液不同组分中SCCPs和MCCPs的回收率、线性范围以及方法检出限,以验证该方法的有效性。以空白样品的3倍标准偏差计算方法检出限,SCCPs和MCCPs的方法检出限分别为1.57 ng/g湿重(ww,*n*=7)和8.29 ng/g ww(*n*=7);提取内标的回收率分别为67.0%~126.6%和69.5%~120.5%,并在所有结果中进行回收率校正。

### 2.5 实际样品分析

采集大连地区孕妇人群血液样品,并将所建立方法应用于人体血液中4种不同组分的分离,经过提取净化后用GC-ECNI-LRMS定量分析SCCPs和MCCPs的水平。结果表明,SCCPs和MCCPs在所有红细胞、血浆、白细胞、血小板中均被检出,相关数据见表3。SCCPs和MCCPs在红细胞中的含量最高,分别为34.27~65.23 ng/g ww和86.80~105.65 ng/g ww;其次为血浆,血浆中SCCPs和MCCPs的平均含量分别为45.59 ng/g ww和64.38 ng/g ww;白细胞和血小板中SCCPs和MCCPs的含量相对较低,其中SCCPs在白细胞和血小板中的含量分别为19.00~32.75 ng/g ww和10.81~22.71 ng/g ww, MCCPs与SCCPs的分布模式相似,其含量最低为31.82 ng/g ww。SCCPs和MCCPs在人体血液红细胞、血浆、白细胞、血小板中的含量分配见图3。由图3可知,SCCPs和MCCPs主要分布于红细胞,其次为血浆,二者在红细胞和血浆中的含量之和分别为70%和66%。在血液不同组分中,MCCPs的含量均高于SCCPs,二者含量的比值为1.04~3.78。

**表3:** 人体血液样品不同组分中SCCPs和MCCPs的含量

Sample No.	Blood component	SCCPs			MCCPs	
		Content/ (ng/g ww)	Recovery/ %		Content/ (ng/g ww)	Recovery/ %
1	RBC	65.23	68.3		105.65	110.5
	plasma	59.01	107.6		61.50	76.5
	WBC	19.00	99.3		31.82	119.9
	platelet	22.71	76.4		37.64	106.9
	sum	165.95	-		236.61	-
2	RBC	51.58	126.6		86.80	111.5
	plasma	47.13	106.5		66.83	72.5
	WBC	32.75	67.0		41.04	69.5
	platelet	10.81	103.1		40.81	102.8
	sum	142.27	-		235.48	-
3	RBC	34.27	96.3		104.53	79.6
	plasma	30.62	86.0		64.80	80.4
	WBC	23.14	89.1		46.17	120.5
	platelet	13.94	97.5		47.99	109.6
	sum	101.97	-		263.49	-

Sum: the total contents of SCCPs or MCCPs for the four blood components in samples; ww: wet weight; -: no value.

**图3:**
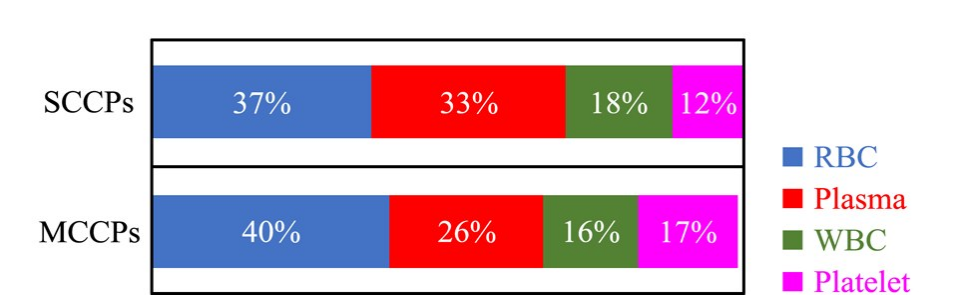
SCCPs和MCCPs在人体血液红细胞、血浆、白细胞、 血小板中的含量分配

通过血液4种组分中SCCPs和MCCPs的含量可以计算出所采集大连地区孕妇血液样品中SCCPs的总含量,分别为165.95、142.27、101.97 ng/g ww, MCCPs总含量分别为236.61、235.48、263.49 ng/g ww,样品间无明显差异。表4总结了国内外不同城市人群血液样品中SCCPs和MCCPs的水平,其中国内城市人体血液中SCCPs和MCCPs的水平明显高于捷克^[19]^;与国内其他城市相比,本实验采集的大连地区孕妇血液样品中的SCCPs和MCCPs处于中等水平^[2,16]^。

**表4:** 国内外不同城市人体血液样品中SCCPs和MCCPs的水平(湿重)对比

City and country	Sample types	Number of samples	SCCP level				MCCP level			Ref.
			Range/(ng/g)		Median/(ng/g)		Range/(ng/g)		Median/(ng/g)	
Dalian, China	RBC, WBC,	3	101.97-	165.95	-		236.48-	263.49	-	this study
	plasma, platelet									
Shenzhen, China	whole blood	50	14-	3500	98		6.3-	320	21	[2]
Guangzhou, China	serum	24	1.00-	5.45^*^	1.7^*^		1.00-	2.74^*^	1.6^*^	[15]
Jinan, China	serum	145	24.2-	384	107		23.8-	371	134	[16]
Prague, Ostrava, Ceske	serum	27	0.9-	13.9	2.2		1.2-	8.3	1.9	[19]
and Budejovice, Czech										

* mass concentration, ng/mL; -: not detected.

SCCPs和MCCPs在人体血液红细胞、血浆、白细胞、血小板中的同族体分布如图4所示。SCCPs在人体血液4种组分中的分布模式相似,均以C_10_-CPs为主,其次是C_11_-CPs、C_12_-CPs和C_13_-CPs;在不同氯取代分布中,Cl_7_组分的相对丰度最高,其次是Cl_6_和Cl_8_组分。MCCPs同族体分布以C_14_-CPs为主,其次是C_15_-CPs和C_16_-CPs;在不同氯取代分布中,Cl_6_组分的相对丰度最高。本实验中SCCPs的碳同族体分布模式与捷克人体血清中的碳同族体分布模式相似^[19]^,氯同族体分布模式与中国济南人群血浆样品相似^[16]^; MCCP的碳同族体分布模式与中国济南^[16]^、挪威北部^[20]^和捷克^[19]^人群的血清样品一致。中国济南和广州人群的血清样品中,C_13_-CPs是相对丰度最高的组分^[15,16]^,与本实验结果存在差异,可能是不同地区CPs生产、使用模式及人群饮食习惯的差异造成的。

**图4:**
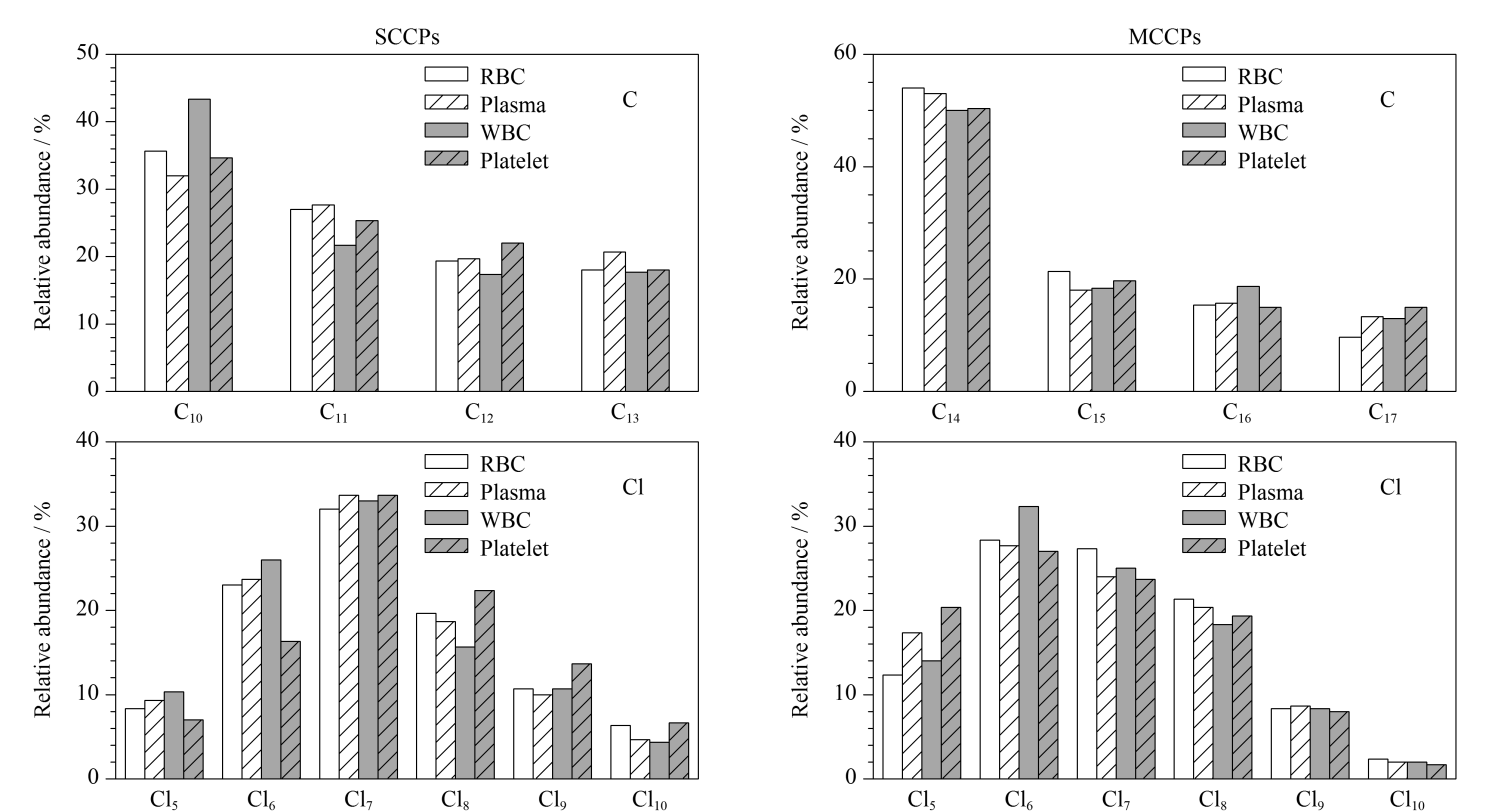
人体红细胞、血浆、白细胞、血小板中SCCPs和MCCPs的同族体分布模式

## 3 结论

本研究以不连续密度梯度的Percoll氯化钠缓冲溶液为分离介质,对人体血液进行离心富集,获得血液不同组分,建立了人体血液的快速相分离技术。此分离技术具有所需样品量小、样品富集操作简单、分离速度快等优点。基于此分离技术,采用超声提取、多层硅胶柱净化和GC-ECNI-LRMS分析检测人体血液不同组分中(血浆、血小板、白细胞、红细胞)SCCPs和MCCPs的水平及分布特征。所建立的方法灵敏度和准确度较高,具有较低的方法检出限,并有望对开展CPs人体内暴露风险评估研究发挥重要作用。
